# Analysis of Genes Involved in Ulcerative Colitis Activity and Tumorigenesis Through Systematic Mining of Gene Co-expression Networks

**DOI:** 10.3389/fphys.2019.00662

**Published:** 2019-05-31

**Authors:** Wanting Shi, Rongjun Zou, Minglei Yang, Lei Mai, Jiangnan Ren, Jialing Wen, Zhaoshi Liu, Renxu Lai

**Affiliations:** ^1^Department of Gastroenterology, Fifth Affiliated Hospital, Sun Yat-sen University, Zhuhai, China; ^2^Digestive Endoscopy Center, Fifth Affiliated Hospital, Sun Yat-sen University, Zhuhai, China; ^3^Department of Cardiovascular Surgery, Sun Yat-sen Memorial Hospital, Sun Yat-sen University, Guangzhou, China; ^4^Department of Genetics, Zhongshan School of Medicine, Sun Yat-sen University, Guangzhou, China; ^5^Guangdong Institute of Gastroenterology, Guangdong, China; ^6^Guangdong Provincial Key Laboratory of Colorectal and Pelvic Floor Diseases, The Sixth Affiliated Hospital, Sun Yat-sen University, Guangzhou, China

**Keywords:** ulcerative colitis, colorectal cancer, pathway enrichment, molecular mechanism, bioinformatics analysis

## Abstract

Ulcerative colitis (UC) is an idiopathic, chronic inflammatory disorder of the colon, characterized by continuous mucosal inflammation. Recently, some studies have considered it as part of an inflammatory bowel disease-based global network. Herein, with the aim of identifying the underlying potential genetic mechanisms involved in the development of UC, multiple algorithms for weighted correlation network analysis (WGCNA), principal component analysis (PCA), and linear models for microarray data algorithm (LIMMA) were used to identify the hub genes. The map of platelet activation, ligand-receptor interaction, calcium signaling pathway, and cAMP signaling pathway showed significant links with UC development, and the hub genes *CCR7*, *CXCL10*, *CXCL9*, *IDO1*, *MMP9*, and *VCAM1*, which are associated with immune dysregulation and tumorigenesis in biological function, were found by multiple powerful bioinformatics methods. Analysis of The Cancer Genome Atlas (TCGA) also showed that the low expression of *CCR7*, *CXCL10*, *CXCL9*, and *MMP9* may be correlated with a poor prognosis of overall survival (OS) in colorectal cancer (CRC) patients (all *p* < 0.05), while no significance detected in both of *IDO1* and *VCAM1*. In addition, low expression of *CCR7*, *CXCL10*, *CXCL9*, *MMP9*, and *IDO1* may be associated with a poor prognosis in recurrence free survival (RFS) time (all *p* < 0.05), but no significant difference was identified in *VCAM1*. Moreover, the *NFKB1*, *FLI1*, and *STAT1* with the highest enrichment score were detected as the master regulators of hub genes. In summary, these results indicated the central role of the hub genes of *CCR7*, *CXCL10*, *CXCL9*, *IDO1*, *VCAM1*, and *MMP9*, in response to UC progression, as well as the development of UC to CRC, thus shedding light on the molecular mechanisms involved and assisting with drug target validation.

## Introduction

Ulcerative colitis (UC) is a global, progressive and complex disease, the incidence of which is still growing, according to large-scale epidemiological statistics studies (Ng et al., 2018). Epidemiological reports shows that the highest annual incidence of UC was 24.3 per 100,000 person-years in Europe, 6.3 per 100,000 person-years in Asia and the Middle East, and 19.2 per 100,000 person-years in North America; and adjusted prevalences have exceeded 0.3% in many countries, especially in Europe and North America (Molodecky et al., 2012; Ng et al., 2018). A further concern is that the incidence of UC and the widespread use of therapeutic agents are associated with an increased risk of cancer (Biancone et al., 2015). Regarding the pathogenesis of UC, most of the emerging evidence supports the concept of an “inflammatory bowel disease (IBD) interactome,” that is, UC is considered as part of a global disease network, with a complex interplay between host genetics, immunity, and environmental factors (Dabritz and Menheniott, 2014). According to this model, gene–environment interactions have pivotal roles in UC progression and mediate UC-related comorbidities and complications, including colitis-associated cancer (Dabritz and Menheniott, 2014). In the past two decades, novel genotyping and sequencing technologies, including RNA expression profiling, DNA methylation profiling, single-cell DNA analysis, chromatin immunoprecipitation sequencing, and RNA sequencing, have launched the era of genetic diseases; so far, 242 susceptibility loci and over 50 hub genes have been discovered in relation to IBD and various phenotypes (Mirkov et al., 2017). Moon et al. performed deep resequencing of UC-associated genes, showing that genetic variants of rs10035653 in *C5orf55*, rs41417449 in *BTNL2*, rs3117099 in *HCG23*, rs7192 in *HLA-DRA*, rs3744246 in *ORMDL3*, and rs713669 in *IL17REL* were significant (Moon et al., 2018). Hong et al. (2018) performed a *trans*-ethnic meta-analysis based on Asian IBD patients and subsequently identified three novel susceptibility loci at *MYO10-BASP1*, *PPP2R3C/KIAA0391/PSMA6/NFKB1A*, and *LRRK1*; as well as four previously known loci at *NCF4*, *TSPAN32*, *CIITA*, and *VANGL2*. Similarly, Peters et al. (2017) presented a predictive model of immune-related genes and further analyzed the functional and regulatory annotations based on genome-wide association studies. Consequently, a driver set including *DOCK2*, *GPSM3*, *AIF1*, *NCKAP1L*, and *DOK3* was selected, representing a high predictive efficiency in the integrated circuits of genetics, molecular, and clinical traits of IBD (Peters et al., 2017). The candidate biomarkers identification of UC activity and tumorigenesis in prior studies were presented in Table 1.

**Table 1:** The candidate biomarkers identification of ulcerative colitis activity and tumorigenesis in prior studies.

Terms	Investigator	Candidate biomarker
UC activity	Moon et al., 2018	C5orf55, BTNL2, HCG23, and HLA-DRA, et al.
	Hong et al., 2018	MYO10-BASP1, PPP2R3C, KIAA0391, and PSMA6, et al.
	Wu et al., 2014	VCAM1, IL6, IL18, ICAM1, and TNFα
	McNamee et al., 2015	CCR7
	Ciorba et al., 2010	IDO1, STAT1, TLR9, and CD11
UC-associated cancer	Zhang et al., 2017	TNFα, IL1β, IFNγ, IL6, IL17a, IL23a, IL4, and IL12a
	Liu et al., 2014	IL1β, IL6, TNFα, NFκB, and STAT3
	Shukla et al., 2016	CXCL9, CXCL10, CCL5, IL1α, IL6, and TNFα
	Pujada et al., 2017	MMP9 and S100A8
	Thaker et al., 2013	IDO1 and IFNγ
	Xu et al., 2018	CCR7, CCL19 and CD31

In summary, these novels genotyping and sequencing technologies and validated hub gene or susceptibility loci not only confer new regulators of pathophysiology, but also open a new horizon to find drug targets and redefine the disease’s regulatory framework. However, these genetic variants combined only explain one in four cases of UC (Uhlig and Muise, 2017). The results also suggest that: (1) The genetic variants considered as personal pathogenic components cannot be isolated in the gene-environment network; (2) These hub genes show better statistical significance while loss of the functional and regulatory annotations or may play an important part in protein-protein interaction (PPI) networks without statistical power; (3) some of the hub gene information may have been missed, owing to low abundance or small fold change (FC); and (4) disease-based co-expression network analysis may further improve the mining efficiency beyond classical methods (Uhlig and Muise, 2017). Based on the above notes, we may apply linear models for microarray data power differential expression analyses (LIMMA), weighted correlation network analysis (WGCNA), and principal component analysis (PCA) to explore the hub gene regulatory network, using high-throughput gene expression arrays in UC, to further elucidate the molecular mechanisms of gene–environment interactions.

## Materials and Methods

### Materials

Raw expression microarray array (CEL data) from the GSE13367, GSE38713, GSE16879, GSE48958, GSE75214, GSE4183, GSE37283, and GSE31106 datasets were downloaded from Gene Expression Omnibus^1^ (Barrett et al., 2013). Probe annotations and platform information were generated by matching with the GPL6244 (HuGene-1_0-st) Affymetrix Human Gene 1.0 ST Array (Affymetrix, Santa Clara, CA, United States).

In this study, we analyzed the patients with colitis exclusively, no other IBD cases included. Here, GSE48958 and GSE75214 are matching with the GPL6244 (HuGene-1_0-st) Affymetrix Human Gene 1.0 ST Array, while GSE13367, GSE38713, GSE16879, GSE37283, and GSE4183 are pairing with the GPL570 (HG-U133_Plus_2) Affymetrix Human Genome U133 Plus 2.0 Array and the GSE31106 is in line with GPL1261 (Mouse430_2) Affymetrix Mouse Genome 430 2.0 Array. Total RNA extracted from mucosal biopsies was used to analyze mRNA expression via Affymetrix arrays, and corresponding grouping information from each sample was subsequently pooled for further correlation analysis. Statistical analysis was performed with the R (version 3.3.2).

### Data Processing

**FIGURE 1:**
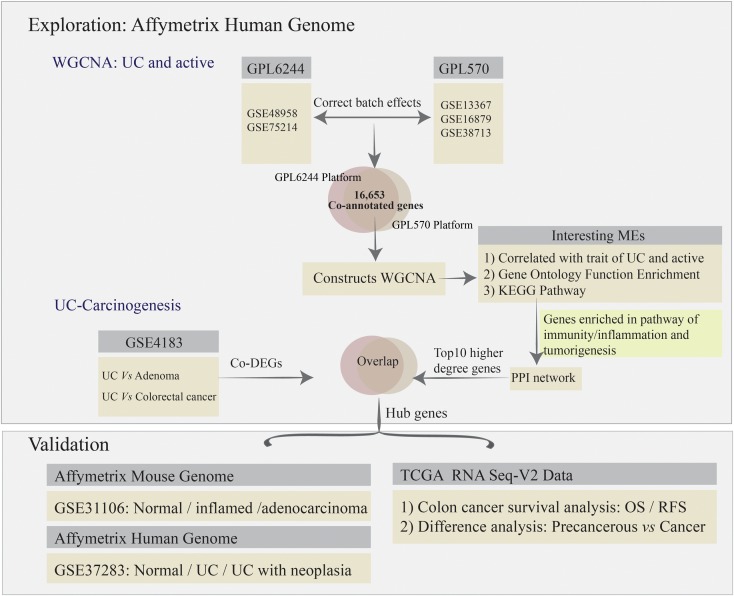
Strategy used for the integrative gene microarray bioinformatics analyses.

To remove bias and variability (resulting from heterogeneity and latent variables) from the high-throughput data for the different microarrays, the “ComBat” function in the SVA package was used to directly adjust the batch effects and latent variables (Leek et al., 2012). Subsequently, all of the microarray raw data analyzed using bioinformatics methods, including background correction, quantile normalization, and probe summarization of the expression values (Irizarry et al., 2003; Ritchie et al., 2015). Some advanced algorithms were used, including: (1) robust multi-array average for background-adjusted, normalized, and log-transformed probe expression values; (2) k-nearest-neighbor for displacing missing values of probes; (3) the *t*-test in the “LIMMA” package to identify differentially expressed genes (DEGs) in mucosal biopsy specimens from the comparative analysis among normal, UC, adenoma, and colorectal cancer (CRC) for GSE4183; and (4) the Benjamini–Hochberg method to adjust *p*-values and thus calculate the false discovery rate and FC (Irizarry et al., 2003; Ritchie et al., 2015). Gene expression values with | log_2_FC| > 1.5 and adjusted *p*-value < 0.05 were used to define DEGs. The co-annotated genes (a total of 16,653 genes) between GPL570 and GPL6244 platform were selected for further co-expression network analysis. The analysis strategy is presented in Figure 1.

### Weighted Co-expression Network Construction and Module Detection

The advantages of co-expression network analysis include the ability to integrate external information and avoid information loss in the case of low-abundance or small-FC genes. Systems-level insight gives WGCNA an edge over other approaches (Langfelder and Horvath, 2008). Therefore, we carried out a systems-level analysis based on WGCNA. The analysis involved the following processes: (1) identifying the appropriate sample basing on the flash-Clust method; (2) selecting a “soft” threshold using the scale-free topology criterion; (3) identifying co-expression modules by employed the dynamic hybrid cut method; (4) relating the co-expression modules to sample traits based on the gene significance (GS) measures, which are defined as the statistical significance of the difference between the gene profile and the sample trait; and (5) accessing the interactions and connectivity of eigengenes among different co-expression modules by the topological overlap matrix method (Langfelder and Horvath, 2008).

### PPI Networks and Functional Enrichment Analysis

We accessed gene biological knowledge, protein functional associations, and PPIs with respect to genetic function, using a web-based analytic tool. The analysis flowchart as flowing that: (1) the gene ontology (GO) functions enrichment was extracted from the DAVID database^2^ (Huang et al., 2007) for annotation, visualization, and integrated discovery bioinformatics resources; GO terms for which *p* < 0.05 were considered to be significantly enriched in the gene modules of interest; and (2) the network of Kyoto Encyclopedia of Genes and Genomes (KEGG) pathway was identified form Metascape database^3^ (Zhou et al., 2019), and *p* < 0.05 and enrichment score >1.0 was set as the cut-off criteria; (3) after carried out for the genes enriched in KEGG pathway of immunity, inflammation and tumorigenesis for the interesting gene modules, we subsequently constructed the PPI biological networks based on the STRING online database (V10.5^4^) with the nodes association confidence score >0.4 (Szklarczyk et al., 2017). In addition, the Cytoscape software (V3.5.1^5^) was used to visualize and evaluate interactions and identifying the hub gene in functional networks (Szklarczyk et al., 2017). The top 10 highest-degree nodes were defined as functional hub genes in the PPI network.

### Identification of Candidate Biomarkers Involving in UC-Associated Carcinogenesis

Additionally, raw data of GSE4183 was used to analysis the co-DEGs involved in UC, adenoma, and CRC. Here, we overlapped the co-DEGs and the PPI network’s functional hub genes, which constructed by WGCNA key modules and identified as important parts in response to the pathway of UC immunity-inflammation and tumorigenesis, to detect the UC-associated carcinogenesis in hub genes. Additionally, the suitable dataset of GSE37283, including the expression profiling of UC with neoplasia, UC and normal mucosa samples, was used to validate the UC-associated carcinogenesis biomarkers; as well as the mouse dataset of GSE31106 involved in the multistep process of “inflammation-dysplasia-cancer.” The human and mouse genes were matched by Gene database^6^ (Brown et al., 2015).

The Cancer Genome Atlas (TCGA) colon adenocarcinoma normalized gene expression value (fragments per kilobase of exon model per million reads mapped, FPKM) were downloaded from the “TCGA biolinks” package (Colaprico et al., 2016). Subsequently, the FPKM data transformed into transcripts per kilobase million (TPM; Li et al., 2010), a comparable data type, which used to apply the survival analysis. The sample and corresponding clinical features were included in further survival and DEGs analysis.

### Investigating the Functional Role and Transcription Factor of Hub Genes

Importantly, the DAVID^7^ and Metascape database were used to explore the GO terms and KEGG pathway enrichment analysis of candidate targets, respectively. The enrichment cut-off criteria keep the same with the chapter and Section “PPI Networks and Functional Enrichment Analysis.” Subsequently, to identify the transcription factor (TF) of the hub genes, the plug in iRegulon for Cytoscape software was applied, with the parameters were set to: (1) minimum identity between orthologous genes = 0.05; (2) maximum FDR for motif similarity = 0.001; and (3) normalized enrichment score (NES) ≥ 3.0 (Janky et al., 2014). Here, the top three regulators with the highest NES value were detected to construct the regulatory network involved in UC-associated carcinogenesis process.

## Results

### Data Processing

**FIGURE 2:**
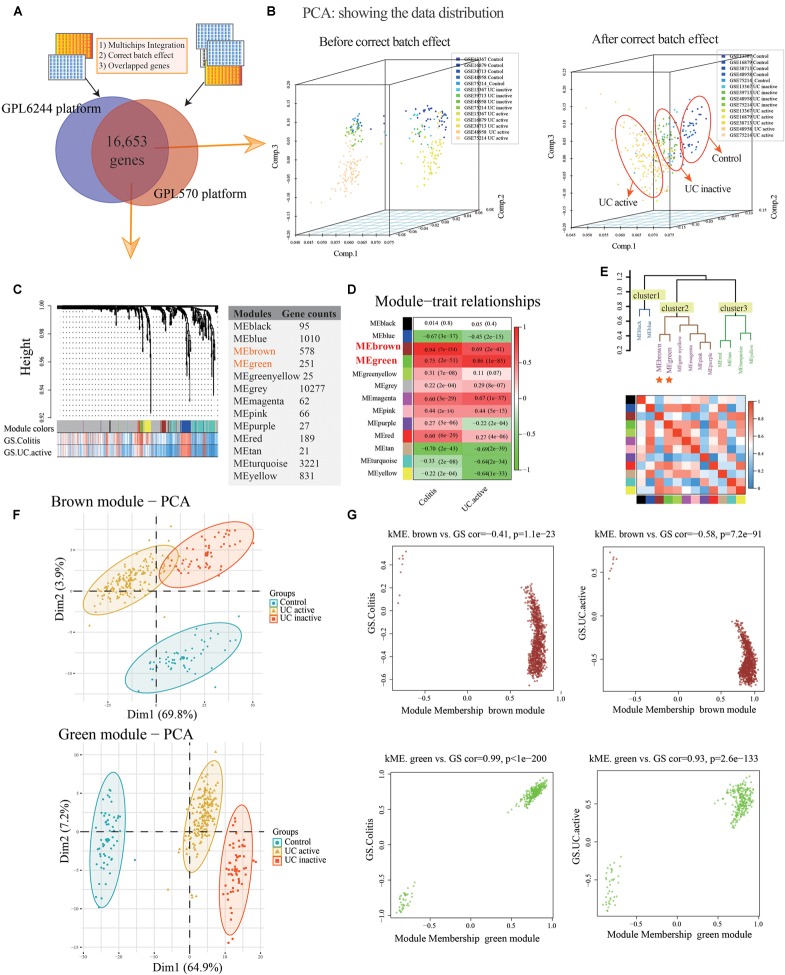
Co-expression modules construction and identify interesting modules of ulcerative colitis and activity. **(A)** Venn diagram showing the overlap of the co-annotated genes in GPL6244 and GPL570 platform. **(B)** The principal component analysis (PCA) for co-annotated genes among the various microarrays in response to pre- and post-adjusting of the batch effects of the status of ulcerative colitis activity. **(C)** Construction of co-expression modules based on a dynamic branch-cutting method. **(D)** The relationship between the co-expression modules and clinical traits. Red represents a positive correlation, and the green represents a negative correlation. **(E)** The connectivity of eigengenes. Red represents a positive correlation, and blue represents a negative correlation. **(F)** The PCA for interesting module genes in response to the status of ulcerative colitis activity. **(G)** The gene significance (GS) and module membership (MM) analysis of interesting modules in response to ulcerative colitis and activity.

The normal and UC mucosa without additional treatment from GPL6244 (GSE48958 and GSE75214) and GPL570 (GSE13367, GSE38713, and GSE16879) platform were selected in their entirety for further analysis, including the 58 normal, 55 UC inactive and 170 UC active samples (Supplementary Table S1). After merging the co-annotated genes, 16,653 genes were retained in further analysis (Figure 2A and Supplementary Table S1). The PCA of co-annotated genes in response to pre- and post-correct the batch effects were showing in Figure 2B, which presenting a significant distinction between control, UC inactive and UC active samples.

### Construction of Co-expression Network and Gene Modules

After sample cluster analysis, the 283 samples with 16,653 gene variables were divided into 13 clusters (MEblack, MEblue, MEbrown, MEgreen, MEgreenyellow, MEgrey, MEmagenta, MEpink, MEpurple, MEred, MEtan, MEturquoise, and MEyellow; Figure 2C), and no samples removed in this process (Supplementary Figure S1A).

**FIGURE 3:**
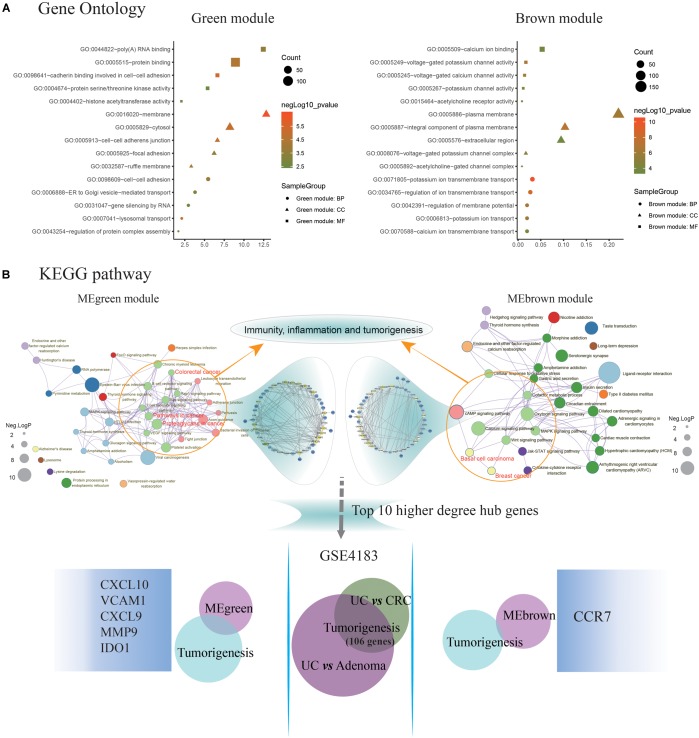
Identification of key pathways and hub genes in the progression of ulcerative colitis activity and tumorigenesis. **(A)** Gene ontology (GO) terms enrichment analyzing was performed using the Database for Annotation, Visualization, and Integration Discovery (DAVID) database. The sizes of the dots represent the counts of enriched module genes, and the dot color represents the negative Log_10_ (*p*-value). **(B)** Kyoto Encyclopedia of Genes and Genomes (KEGG) pathway enrichment was constructed by Metascape database. The sizes of the dots represent the negative Log_10_ (*p*-value). Subsequently, the genes enriched in the pathway of immunity, inflammation, and tumorigenesis were selected to construct the protein-protein interaction (PPI) network basing on the STRING database for modules with a threshold value >0.4, respectively. And the Venn diagram showing the overlap of the top 10 functional hub genes in the MEgreen and MEbrown PPI networks, and tumorigenesis genes detected in GSE4183. The font sizes represent the degree of gene interaction in the PPI network.

Following the WGCNA: (1) when the critical parameter of the power value was 12, the scale independence was up to 0.8 and had a higher mean connectivity (Supplementary Figure S1B); (2) two key modules and the relationship with the clinical traits were detected (Colitis: MEbrown Pearson coefficient = 0.94, *p* = 7E-134, MEgreen Pearson coefficient = 0.75, *p* = 2E-51; UC active: MEbrown Pearson coefficient = 0.69, *p* = 2E-41; MEgreen Pearson coefficient = 0.86, *p* = 1E-85; Figure 2D); (3) the result of interaction analysis among co-expression modules suggested a high degree of independence among different module genes, such that the heatmap showed no significant interaction among module genes (Supplementary Figure S1C); and (4) The connectivity of eigengenes in different modules allowed us to identify three clusters identified, and the eigengenes of different modules within the same cluster showed significant connectivity, whereas there was no difference among different clusters’ modules (Figure 2E). The interesting module gene list was presented in Supplementary Table S2.

Additionally, in related to UC activity, two-dimensional PCA results also showing satisfactory connectivity and distinguish ability of MEgreen and MEbrown module genes in response to UC and activity (Figure 2F; MEgreen: first principal component: 64.9%, second principal component: 7.2%; MEbrown: first principal component: 69.8%, second principal component: 23.9%). And, as shown in Figure 2G, the GS analysis results showing a tight correlation between the gene and the trait of colitis (MEbrown: Pearson coefficient = -0.41, *p* = 1.1E-23; MEgreen: Pearson coefficient = 0.99, *p* < 1E-200), as well as the trait of UC active (MEbrown: Pearson coefficient = -0.58, *p* = 7.2E-91; MEgreen: Pearson coefficient = 0.93, *p* = 2.6E-133). Similarly, the MEbrown and MEgreen module genes also presented a significant contribution to the module membership (MM; Figure 2G).

### Functional Enrichment Analysis and PPI Networks Construction

The MEgreen and MEbrown modules were assessed for further functional enrichment, consisting of GO term enrichment analysis of module genes of interest. Regarding GO terms enrichment, the MEgreen module was mainly enriched in GO: 0007041∼lysosomal transport (5 genes enriched; *p* = 1.11E-05), GO: 0098609∼cell-cell adhesion (13 genes enriched; *p* = 1.68E-04), and GO: 0043254∼regulation of protein complex assembly (4 genes enriched; *p* = 6.43E-04). Genes in the MEbrown module were predominantly enriched in GO: 0071805∼ion transmembrane transport (21 genes enriched; *p* = 3.98E-11), GO: 0042391∼regulation of membrane potential (13 genes enriched; *p* = 4.80E-07), and GO: 0034765∼regulation of ion transmembrane transport (18 genes enriched; *p* = 3.95E-09). These results are illustrated in Figure 3A. Regarding the KEGG pathway enrichment, the MEgreen module genes were significantly enriched in viral carcinogenesis (15 genes enriched; enrichment score = 5.01; *p* = 3.51E-05), proteoglycans in cancer (10 genes enriched; enrichment score = 3.97; *p* = 1.01E-03), and platelet activation (8 genes enriched; enrichment score = 4.91; *p* = 1.46E-03). However, genes in the MEbrown module were significantly enriched in ligand-receptor interaction (22 genes enriched; enrichment score = 3.55; *p* = 3.41E-07), calcium signaling pathway (13 genes enriched; enrichment score = 3.21; *p* = 1.72E-03), and adenosine 3′, 5′-cyclic monophosphate (cAMP) signaling pathway (12 genes enriched; enrichment score = 2.72; *p* = 2.36E-03).

Additionally, the Figure 3B illustrating a part of the visible pathway that tightly correlated with cancer from the KEGG pathway network (Table 2). These cancer-correlated pathways were related to immunity-inflammation response and tumorigenesis. These results also illustrated in Table 2. After submitting the genes enriched in cancer-correlated pathways to the STRING database, 50 and 48 PPI nodes were obtained for the MEbrown and MEgreen modules, respectively, with a confidence threshold greater than 0.4. After analyzed by Cytoscape software as an undirected method, the top 10 highest connectivity nodes of each PPI network were considered to be central agents. The PPI network of interesting modules was presented in Figure 3B.

**Table 2:** Identifying the cancer-correlated terms form the KEGG pathway network of the interesting modules.

MEmodule	KEGG term	*p* Value	Enriched genes
MEgreen	Platelet activation	0.001	AKT2, RHOA, ITGB1, MMP9, PRKACA, et al.
	Proteoglycans in cancer	0.001	AKT2, RHOA, ITGB1, MDM2, PRKACA, et al.
	B cell receptor signaling pathway	0.005	AKT2, PPP3R1, RAC1, IKBKG
	Chronic myeloid leukemia	0.005	AKT2, MDM2, PTPN11, IKBKG
	Pathways in cancer	0.006	AKT2, RHOA, DAPK3, ITGB1, MDM2, et al.
	Bacterial invasion of epithelial cells	0.007	RHOA, RAC1, CXCL10, CXCL9, CXCL11, et al.
	Ras signaling pathway	0.008	AKT2, RHOA, PRKACA, PTPN11, RAC1, et al.
	T cell receptor signaling pathway	0.019	AKT2, RHOA, PPP3R1, IKBKG, CCL19
	Rap1 signaling pathway	0.019	AKT2, RHOA, ITGB1, RAC1, TLN1, et al.
	VEGF signaling pathway	0.021	AKT2, PPP3R1, RAC1, VCAM1, PTPN11
	Colorectal cancer	0.022	AKT2, RHOA, RAC1, CD19, CD274
	Leukocyte transendothelial migration	0.027	RHOA, ITGB1, PTPN11, RAC1
	Adherens junction	0.035	RHOA, PTPN1, RAC1, TNFSF13B, IDO1, et al.
	Pertussis	0.041	RHOA, CFL1, ITGB1, GZMB
Mebrown	Calcium signaling pathway	0.000	ATP2B3, AVPR1B, CCKAR, CACNA1B, CAMK2B, et al.
	cAMP signaling pathway	0.002	ATP2B3, HCN2, CAMK2B, CNGA4, DRD2, et al.
	Oxytocin signaling pathway	0.002	CAMK2B, KCNJ5, OXT, PRKACG, PRKCG, et al.
	MAPK signaling pathway	0.013	CACNA1B, FGF6, MAPT, PRKACG, PRKCG, et al.
	Wnt signaling pathway	0.015	CAMK2B, PRKACG, PRKCG, CCR7, SFRP5, et al.
	Jak-STAT signaling pathway	0.024	CNTFR, EPO, GFAP, GH2, IFNA2, et al.
	Cofactor metabolic process	0.026	CAMK2B, DCT, PRKACG, PRKCG, WNT7B, et al.
	Basal cell carcinoma	0.034	GLI1, WNT7B, WNT8A, CCR7, APC2, et al.
	Cytokine-cytokine receptor interaction	0.041	AMHR2, CNTFR, EPO, GH2, IFNA2, et al.
	Breast cancer	0.043	FGF6, PGR, WNT7B, WNT8A, FGF23, et al.

### Identification of Candidate Biomarkers Involving in UC-Associated Carcinogenesis

**FIGURE 4:**
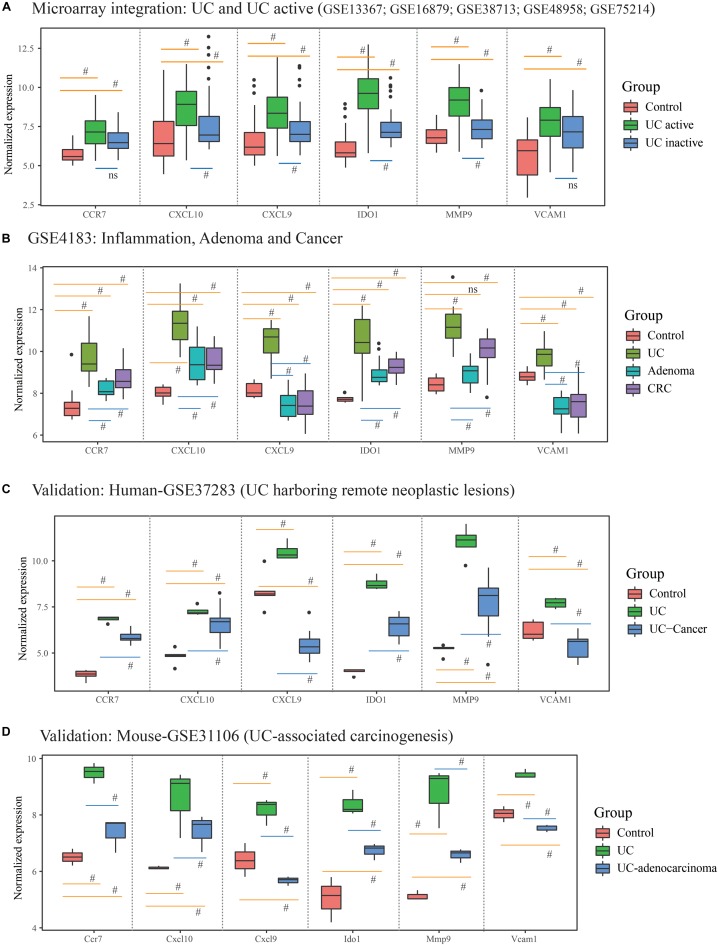
The gene expression analysis and validation in response to the ulcerative colitis activity and tumorigenesis of hub genes. **(A)** The hub gene’s expression in response to ulcerative colitis and activity in integrative microarray datasets. **(B)** The hub gene’s expression detection of GSE4183 involved in inflammation, adenoma and colonic cancer. (**C,D**) The validation in the progression of UC-associated carcinogenesis for six candidate genes in human and mouse microarray datasets. #Represent *p* < 0.05 among the multi-comparison between normal, UC, and adenocarcinoma mucosa.

What’s more, 184 DEGs were obtained in the comparison of UC and CRC (160 DEGs down-regulated and 24 DEGs up-regulated in CRC samples), and 344 DEGs were identified in the comparison of UC and adenoma in GSE4183 (332 DEGs down-regulated and 12 DEGs up-regulated in adenoma samples) (Supplementary Table S3). After overlapped, 106 co-DEGs were selected. Subsequently, we’ve further identified the same genes between co-DEGs and central agents of each PPI network, which have been selected as UC-related tumorigenesis genes. And 6 (*CXCL10*, *VCAM1*, *CXCL9*, *MMP9*, *IDO1*, and *CCR7*) out of the 106 co-DEGs remained after selection (Figure 2B and Supplementary Table S5). We also found a statistical difference in the gene expression levels of these genes between healthy individuals and UC patients (Figure 4A and Supplementary Table S4), as well as the expression levels between healthy individuals, UC, adenoma, and CRC patients of GSE4183 dataset (Figure 4B and Supplementary Table S5).

After validated by GSE37283 and GSE31106 datasets, the six hub gene expression levels in phases of UC were significantly increased in compared with the normal sample in both of the human and mouse’s colonic mucosa (all the *p* < 0.05; Supplementary Table S6). Additionally, in comparison with UC mucosa, the expression level of six hub genes was decreased in phases of adenocarcinoma in human’s colonic mucosa (all the *p* < 0.05; Figure 4C,D). Additionally, in comparison with normal samples, the expression level of *CCR7*, *CXCL10*, *IDO1*, and *MMP9* were increased in phases of adenocarcinoma’s tissue, while the expression level of *CXCL9* and *VCAM1* were decreased. These results are shown in Figure 4 and Supplementary Table S6.

**FIGURE 5:**
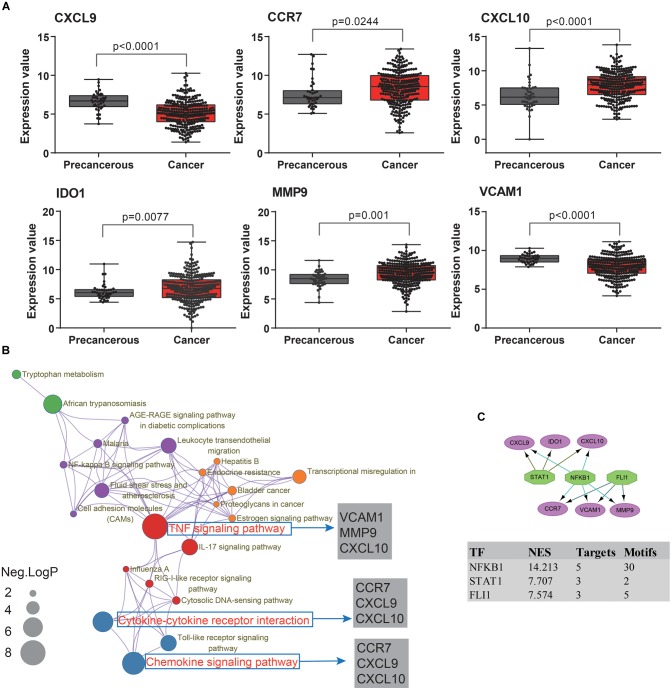
The molecular mechanism and transcription factors investigation for candidate biomarkers involved in ulcerative colitis activity and tumorigenesis. **(A)** The hub gene’s expression levels analysis among cancer and para-cancerous tissues. **(B)** The KEGG pathway enrichment analysis of detected hub genes based on Metascape database. The sizes of the dots represent the negative Log_10_ (*p*-value). **(C)** The regulatory network results, based on the iRegulon plugin, showing that the top three regulators of *NFKB1*, *STAT1*, and *FLI1* in a set of six hub genes, were selected with the highest normalized enrichment score (NES).

**FIGURE 6:**
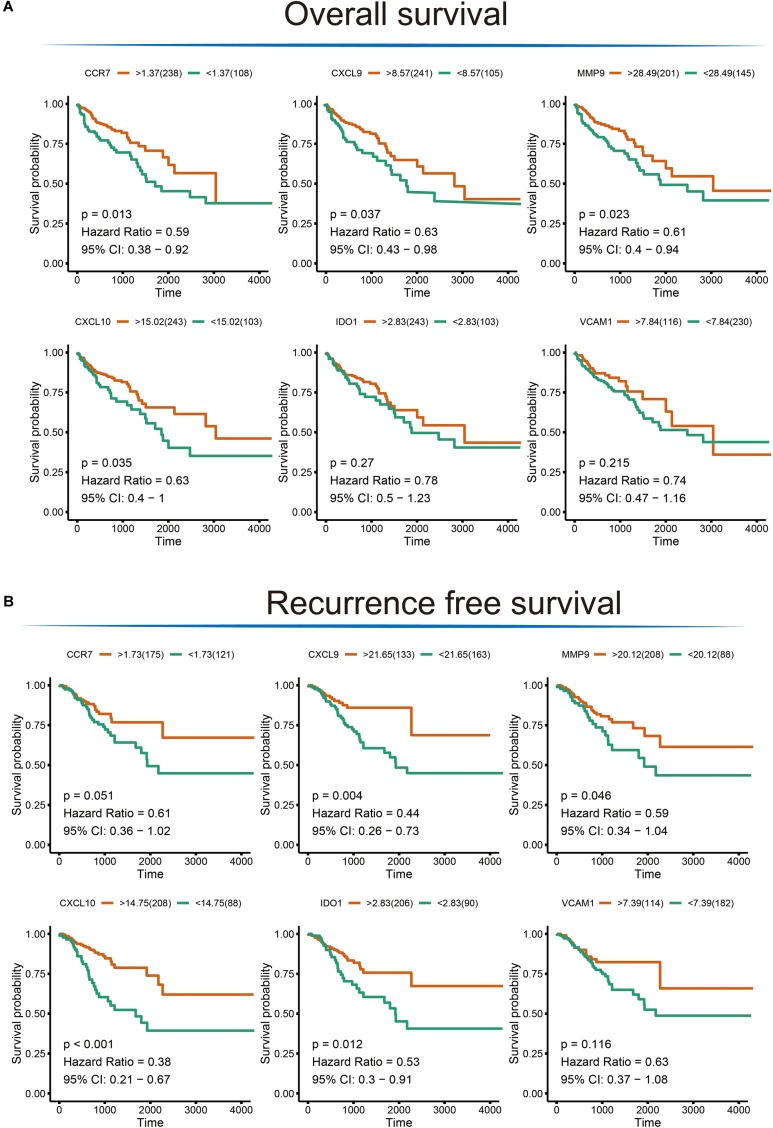
The Kaplan–Meier survival analysis for the hub genes. **(A)** Overall survival analysis illustrating the significant difference between higher expression level and lower expression level of *CXCL10*, *CXCL9*, *MMP9*, and *CCR7* gene with respect to patient survival time; no difference was observed for the *VCAM1* and *IDO1* gene. **(B)** Significant differences were detected for the *CXCL10*, *CXCL9*, *MMP9*, *IDO1*, and *CCR7* in recurrence free survival time; no difference was observed for the *VCAM1* gene.

To extend our findings, the gene expression levels in CRC and para-cancerous tissues were compared based on TCGA database. Consequently, the gene differential expression level with regrading to hub genes was constructed; and all of the hub genes shown a significant difference in expression level between cancer and para-cancer tissues (all the *p* < 0.05; Figure 5A). Importantly, the Kaplan–Meier survival curves indicated that a higher expression level of *CXCL10* (hazard ratio = 0.63; *p* = 0.035), *CXCL9* (hazard ratio = 0.63; *p* = 0.037), *MMP9* (Hazard Ratio = 0.61; *p* = 0.023), and *CCR7* (Hazard Ratio = 0.59; *p* = 0.013) were significantly associated with the poor prognosis for CRC patients; although the difference in overall survival (OS) between high and low expression of *IDO1* (Hazard Ratio = 0.78; *p* = 0.27) and *VCAM1* (Hazard Ratio = 0.74; *p* = 0.215) were not significant. Additionally, there was a clear tendency for lower expression of *CXCL10* (Hazard Ratio = 0.38; *p* < 0.001), *CXCL9* (Hazard Ratio = 0.11; *p* = 0.004), *MMP9* (Hazard Ratio = 0.59; *p* = 0.046), *IDO1* (Hazard Ratio = 0.53; *p* = 0.012) and *CCR7* (Hazard Ratio = 0.61; *p* = 0.051) to be associated with a better prognosis in the recurrence free survival (RFS) time. This suggests that, to some extent, the effect of *CXCL10*, *CXCL9*, *MMP9*, *IDO1*, and *CCR7* overexpression on early survival time resulted in a decrease in the survival rate. And these results are shown in Figure 6.

### Investigating the Functional Role and TF of Hub Genes

To further understand how the hub genes were correlated with UC-associated carcinogenesis, we applied DAVID and Metascape online database to explore the biological function. The results of GO term enrichment indicated that the GO:0032496∼response to lipopolysaccharide (Enriched genes: *CCR7*, *CXCL9*, *IDO1*, *CXCL10*; *p* = 1.87E-06), GO:0030816∼regulation of cAMP metabolic process (Enriched genes: *CXCL9*, *CXCL10*; *p* = 1.25E-03), and GO:0006954∼inflammatory/immune response (Enriched genes: *CCR7*, *CXCL9*, *CXCL10*; *p* = 2.11E-03) were mainly enriched, while pathway of Ecb04668: TNF signaling pathway (Enriched genes: *CXCL10*, *VCAM1*, *MMP9*; *p* = 1.36E-03), Ecb04062: Chemokine signaling pathway (enriched genes: *CCR7*, *CXCL9*, *CXCL10*; *p* = 3.76E-03), and Ecb04060: Cytokine-cytokine receptor interaction (enriched genes: *CCR7*, *CXCL9*, *CXCL10*; *p* = 4.64E-03) (Figure 5B).

Finally, we predicted TFs and found that nuclear factor NF-kappa-B1 (*NFKB1*) (NES = 14.21, target genes = 5, motifs = 30), friend leukemia integration 1 TF (*FLI1*) (NES = 7.57, target genes = 3, motifs = 5), and signal transducer and activator of transcription 1 (*STAT1*) (NES = 7.71, target genes = 3, motifs = 2) as the master regulators of the hub genes are involved in UC-associated carcinogenesis (Figure 5C).

## Discussion

After adjusting the batch effects, 16,653 co-annotated genes among GPL6244 and GPL570 platform microarray datasets. Subsequently, we included co-annotated genes, some of which were present in low abundance or with small FC, in a further analysis, in which combination with WGCNA could integrate external traits and avoid information loss at a system level. According to the results, both MEbrown and MEgreen appeared to be moderately effective in revealing the UC-based global network. Biologically, following the functional enrichment analysis, the pathways of viral carcinogenesis, proteoglycans in cancer, platelet activation, ligand-receptor interaction, calcium signaling pathway, and cAMP signaling pathway were identified as being significantly associated with UC active. And cancer with highly correlated pathway and enriched genes were selected to construct the PPI network. The most critical genes were *CXCL10*, *VCAM1*, *CXCL9*, *MMP9*, *IDO1*, and *CCR7*, indicating that genetic variability influences susceptibility to the disease global network, and subsequently revealing potential regulatory roles in UC-associated carcinogenesis. Furthermore, these hub genes majorly enriched in tumor necrosis factor (TNF) signaling pathway, chemokine signaling pathway, and cytokine-cytokine receptor interaction; and potentially regulated by *NFKB1*, *FLI1*, and *STAT1* in TFs network analysis. Furthermore, a significant association of *CCR7*, *CXCL10*, *CXCL9*, *IDO1*, and *MMP9* with UC-correlated CRC development was identified by integrating gene expression and survival analysis.

Emerging evidence has revealed the central role of gene-environment interaction in UC-based disease networks. Extrinsic and intrinsic environmental factors may cause chronic or acute inflammation in UC patients. Wang et al. found that calcium signaling pathway contributes to the development of colonic dysmotility in UC and intestinal inflammation, may be related with the calcium-transporting ATPase dysregulation in epithelial cells (Wang et al., 2016). And, additionally, the evidence for the interdependence of platelet abnormalities in UC model and patients, suggesting that the pathological state of changes in platelet parameters and their activation, may be linked to the inflammatory response and enhanced platelet-leukocyte, and aggregate formation associated with colitis (Senchenkova et al., 2015; Gawronska et al., 2017). Proteoglycans have been found to be critical in the regulation of stem cell through inducing precise and coordinated modulation of key growth factors, resulting in selective mitogen-activated protein kinases (MAPK) and/or another intracellular signaling, demonstrating an aberrant expression of ligand-receptor interaction on immune cells in IBD patients (Elshal et al., 2016; Gawronska et al., 2017). Li et al. demonstrated the multiple proinflammatory signaling pathways and candidate biomarkers, including *STAT1*, *STAT6*, and cAMP signaling pathway, in the exacerbation of UC (Li et al., 2012). Boothello et al. (2019) revealed that proteoglycans mediate cancer stem cells induced CRC xenograft’s growth in a dose-dependent fashion. Moreover, syndecan-2, a type of proteoglycan, up-regulates MMP-7 expression in colon cancer cells via PKCγ-mediated activation of FAK/ERK signaling (Jang et al., 2017). Therefore, the pathway of the calcium signaling pathway, ligand-receptor interaction, platelet activation, cAMP signaling pathway and the none-cancer pathway involved, may provide insight into the immunological and inflammatory response, and the hypothesis of phospholipid-related barrier defects in the intestinal mucosa offers an opportunity to further understand UC-based pathogenesis.

The association between UC-related chronic inflammation and colon cancer has long been recognized (Table 1). According to a systemic review reported by Tatiya-Aphiradee, the pathway immuno-inflammatory response was closely linked to the regulation and maintenance of UC pathogenesis, that directly mediated by dynamic and complex communication between immune cells and cytokines (Tatiya-Aphiradee et al., 2018). Biologically, the dysregulation of antigen recognition, neutrophil chemotaxis, commensal microflora, and epithelial barrier defects may provide insight into the immunological and inflammatory response, it might offer an opportunity to further understand UC-based pathogenesis (Hindryckx et al., 2016). Gene expression profiling by Zhang’s group shown the pathways includes PI3K-Akt signaling, cytokine-cytokine receptor interaction and ECM-receptor interaction was significantly associated with the process of colitis-associated carcinogenesis (Zhang et al., 2017). Several potential biomarkers of TNF signaling pathway, including *TNF-α*, *IL-6*, *IL-1*, *TGF-β*, and *IL-10*, have been confirmed to be involved in the process of malignant transformation of cells and carcinogenesis (Zhang et al., 2017). The pathway of cytokine-cytokine receptor interaction may also be closely linked to UC-related inflammation and tumorigenesis. Fang et al. (2015) compared IBD microarray datasets and found an important role for cytokine-cytokine receptor pathway dysregulation in both pediatric and mouse models of colitis. In sum, during the procession of intestinal inflammation and carcinogenesis, a variety of immunological and inflammatory signaling events, including the TNF signaling pathway, chemokine signaling pathway, and cytokine-cytokine receptor interaction, are activated and involved in a complex biological process.

Among the candidate biomarkers, the current understanding of the function of *CXCL10* and *CXCL9* may recruit the leukocytes to inflammation sites. However, a novel report from Shukla’s group demonstrated that both *CXCL10* and *CXCL9* may promote colonic tumorigenesis via promotes the cytokine-mediated mucosal injury and inflammation response (Shukla et al., 2016). Additionally, *IDO1* were over-expressed in inflamed and adenoma of the colon, also functioned in promotes colitis-associated tumorigenesis independent of T-cell immune surveillance (Thaker et al., 2013). *MMP9* could maintain the microbiota and colonic epithelium mucosal barrier, also correlated with tissue remodeling and carcinogenesis via activates the EGFR signaling pathway (Pujada et al., 2017). The adhesion molecules VCAM-1 and ICAM-1, associated with macrophage infiltration, are directly associated with cell transmigration in inflamed colonic tissue (Wu et al., 2014). In addition, Bernhard et al. revealed that VCAM1 was correlated with different subsets of three immune cells and with high densities of T-cell subpopulations within specific tumor regions in CRC, thus the expression of adhesion molecules also associated with survival prognosis (Mlecnik et al., 2010). What’s more, the lymphoid chemokine receptor CCR7 was re-expressed by activated T cells, allowing them to flow from the tissue to the lymph nodes through afferent lymphatics. McNamee’s data showed a critical role for CCR7 in orchestrating immune cell traffic (McNamee et al., 2015). The role of chemokines in tumor angiogenesis was achieved in a CCR7-dependent manner through inhibiting Met/ERK/Elk-1/HIF-1α/VEGF-A pathway in CRC (Xu et al., 2018).

Finally, the TFs analysis results shown that *NFKB1*, *FLI1*, and *STAT1* were significantly predicted in hub gene’s regulatory network, correlated with UC-correlated tumorigenesis. Here, *STAT1*, the first member of signal transducer and activator of transcription (STAT) family, has been involved in cancer suppression, including CRC (Zamanian-Azodi and Rezaei-Tavirani, 2019). Schwiebs et al. (2019) found that *STAT1* has involved in the process of tumor immune microenvironment during the crosstalk of “inflammation-to-tumor.” NF-kappa-B1 (*NF-κB1*) signaling is a prominent and widely studied inflammatory signaling cascade in the field of immunology (Eden et al., 2017). Increased transcription of NF-κB is associated with inflammation and angiogenesis. Burkitt proposed that *NF-κB1* differentially regulate susceptibility to colitis-associated adenoma development (Burkitt et al., 2015). *FLI1*, a member of the family of ETS TFs, contains a highly conserved domain that recognizes ETS core consensus sites (GGAA/T; Hollenhorst et al., 2011; Tang et al., 2015). EWS-FLI1 regulates multiple target genes through binding to typical ETS core consensus sites or GGAA microsatellites, then participates in the carcinogenic process (Lessnick and Ladanyi, 2012; Tang et al., 2015). Azuara et al. (2018) have defined *FLI1* as a DNA methylation signature that can be distinguished in the early detection of CRC associated with IBD.

In summary, we found that the pathways of platelet activation, ligand-receptor interaction, calcium signaling pathway, and cAMP signaling pathway may play an important role in UC development via multiple physiological and pathophysiological processes, revealing a potentially attractive therapeutic target for UC-based disease networks. The overlapping results for *CXCL10*, *VCAM1*, *CXCL9*, *MMP9*, *IDO1*, and *CCR7* were obtained, which are considered to be hub biomarkers involved in UC-correlated tumorigenesis. Following the expression validation, survival analysis, and functional analysis, our results indicated that the novel biomarkers of *CXCL10*, *VCAM1*, *CXCL9*, *MMP9*, *IDO1*, and *CCR7* has powerful statistical efficiency and biological function. These genes are also linked to immune dysregulation and inflammation response, and thus provide new insights into the pathogenetic mechanisms of UC development and tumorigenic processes. Finally, our results also subsequently identified that the master regulators of *NFKB1*, *FLI1*, and *STAT1* have significantly associated with UC activity and carcinogenesis via target the candidate biomarkers.

## Author Contributions

WS, RZ, and MY took the responsibility for all aspects of the reliability and freedom from bias of the data presented and their discussed interpretation, and drafting the article. LM, JR, ZL, and JW took the responsibility for statistical analyses and interpretation of data. RL took the responsibility for full-text evaluation and guidance, and final approval of the version to be submitted.

## Conflict of Interest Statement

The authors declare that the research was conducted in the absence of any commercial or financial relationships that could be construed as a potential conflict of interest.

## Footnotes

1 http://ncbi.nlm.nih.gov/geo/

2 http://david.abcc.ncifcrf.gov/

3 http://metascape.org/

4 http://string-db.org/

5 http://cytoscape.org/

6 https://www.ncbi.nlm.nih.gov/gene/

7 http://david.abcc.ncifcrf.gov/
