# Supplementary material for: Analysis of Genes Involved in Ulcerative Colitis Activity and Tumorigenesis Through Systematic Mining of Gene Co-expression Networks
Source: Front Physiol. 2019 May 31;10:662. doi: 10.3389/fphys.2019.00662 (PMC6554330; doi:10.3389/fphys.2019.00662)
Supplement: Supplementary file 4 [file Table_4.doc]

**Table S4**. The expression level of 6 hub genes for the UC and activity.

|  | **Control** | **UC inactive** | **UC active** | **Control vs UC inactive** | | **Control vs UC active** | | **UC inactive vs UC active** | |
| --- | --- | --- | --- | --- | --- | --- | --- | --- | --- |
|  | (Mean±SE) | (Mean±SE) | (Mean±SE) | 95% CI | Adj. P | 95% CI | Adj. P | 95% CI | Adj. P |
| ***CCR7*** | 5.72±0.07 | 6.67±0.11 | 7.20±0.07 | -1.33 to -0.57 | < 0.0001 | -1.78 to -1.17 | < 0.0001 | -0.84 to -0.22 | 0.0002 |
| ***CXCL10*** | 6.75±0.2 | 7.65±0.23 | 8.76±0.10 | -1.57 to -0.24 | 0.004 | -2.54 to -1.47 | < 0.0001 | -1.65 to -0.56 | < 0.0001 |
| ***CXCL9*** | 6.60±0.17 | 7.43±0.17 | 8.47±0.11 | -1.43 to -0.22 | 0.0043 | -2.35 to -1.38 | < 0.0001 | -1.54 to -0.54 | < 0.0001 |
| ***VCAM1*** | 5.70±0.17 | 7.18±0.16 | 7.76±0.09 | -2.03 to -0.94 | < 0.0001 | -2.50 to -1.62 | < 0.0001 | -1.03 to -0.13 | 0.0078 |
| ***MMP9*** | 6.90±0.08 | 7.41±0.11 | 9.04±0.09 | -0.97 to -0.05 | 0.0249 | -2.52 to -1.77 | < 0.0001 | -2.01 to -1.25 | < 0.0001 |
| ***IDO1*** | 6.11±0.11 | 7.56±0.17 | 9.58±0.12 | -2.05 to -0.84 | < 0.0001 | -3.95 to -2.98 | < 0.0001 | -2.52 to -1.52 | < 0.0001 |
