# Supplementary material for: Analysis of Genes Involved in Ulcerative Colitis Activity and Tumorigenesis Through Systematic Mining of Gene Co-expression Networks
Source: Front Physiol. 2019 May 31;10:662. doi: 10.3389/fphys.2019.00662 (PMC6554330; doi:10.3389/fphys.2019.00662)
Supplement: Supplementary file 5 [file Table_5.doc]

**Table S5**. The expression level of colitis-associated tumorigenesis biomarkers in GSE4183.

|  | Control | UC | Adenoma | CRC | Control vs UC | |
| --- | --- | --- | --- | --- | --- | --- |
|  | (Mean±SE) | (Mean±SE) | (Mean±SE) | (Mean±SE) | 95% CI | Adj. P |
| *CCR7* | 7.57±0.36 | 9.78±0.26 | 8.20±0.10 | 8.70±0.18 | -3.13 to -1.30 | < 0.0001 |
| *CXCL10* | 8.03±0.12 | 11.24±0.25 | 9.51±0.24 | 9.60±0.18 | -4.17 to -2.26 | < 0.0001 |
| *CXCL9* | 8.14±0.13 | 10.47±0.28 | 7.50±0.16 | 7.50±0.21 | -3.28 to -1.38 | < 0.0001 |
| *VCAM1* | 8.81±0.11 | 9.76±0.18 | 7.30±0.15 | 7.50±0.20 | -1.73 to -0.178 | 0.0103 |
| *MMP9* | 8.44±0.14 | 11.24±0.25 | 8.92±0.15 | 10.15±0.34 | -3.89 to -1.72 | < 0.0001 |
| *IDO1* | 7.73±0.06 | 10.40±0.37 | 8.98±0.16 | 9.25±0.12 | -3.70 to -1.65 | < 0.0001 |

|  | Control vs. Adenoma | | Control vs. CRC | | UC vs. Adenoma | | UC vs. CRC | |
| --- | --- | --- | --- | --- | --- | --- | --- | --- |
|  | 95% CI | Adj. P | 95% CI | Adj. P | 95% CI | Adj. P | 95% CI | Adj. P |
| *CCR7* | -1.55 to 0.29 | 0.027 | -2.05 to -0.21 | 0.01 | 0.82 to 2.35 | < 0.0001 | 0.31 to 1.84 | 0.0025 |
| *CXCL10* | -2.43 to -0.52 | 0.0008 | -2.53 to -0.62 | 0.0004 | 0.94 to 2.53 | < 0.0001 | 0.85 to 2.44 | < 0.0001 |
| *CXCL9* | -0.30 to 1.60 | 0.028 | -0.30 to 1.60 | 0.028 | 2.19 to 3.77 | < 0.0001 | 2.18 to 3.77 | < 0.0001 |
| *VCAM1* | 0.73 to 2.28 | < 0.0001 | 0.53 to 2.08 | 0.0002 | 1.81 to 3.10 | < 0.0001 | 1.61 to 2.90 | < 0.0001 |
| *MMP9* | -1.57 to 0.60 | 0.63 | -2.80 to -0.63 | 0.0006 | 1.41 to 3.22 | < 0.0001 | 0.19 to 1.99 | 0.0125 |
| *IDO1* | -2.27 to -0.22 | 0.011 | -2.54 to -0.49 | 0.0014 | 0.57 to 2.28 | 0.0003 | 0.30 to 2.01 | 0.0041 |
