# Supplementary material for: Analysis of Genes Involved in Ulcerative Colitis Activity and Tumorigenesis Through Systematic Mining of Gene Co-expression Networks
Source: Front Physiol. 2019 May 31;10:662. doi: 10.3389/fphys.2019.00662 (PMC6554330; doi:10.3389/fphys.2019.00662)
Supplement: Supplementary file 6 [file Table_6.doc]

**Table S6**. The expression level validation of 6 hub genes for the different phases of UC-to-cancer.

S5-Table 1. The expression level of 6 hub genes for the different phases of UC-related carcinogenesis in GSE37283.

|  | Control | UC | UC-Cancer | Control vs UC | | Control vs UC-Cancer | | UC vs UC-Cancer | |
| --- | --- | --- | --- | --- | --- | --- | --- | --- | --- |
|  | (Mean±SE) | (Mean±SE) | (Mean±SE) | 95% CI | Adj. P | 95% CI | Adj. P | 95% CI | Adj. P |
| *CCR7* | 3.81±0.13 | 6.84±0.09 | 5.85±0.09 | -3.51 to -2.54 | < 0.0001 | -2.43 to -1.65 | < 0.0001 | 0.57 to 1.41 | < 0.0001 |
| *CXCL10* | 4.82±0.19 | 7.28±0.14 | 6.64±0.28 | -3.73 to -1.19 | 0.0003 | -2.84 to -0.80 | 0.0008 | 0.47 to 1.75 | 0.03243 |
| *CXCL9* | 8.38±0.45 | 10.50±0.25 | 5.49±0.23 | -3.47 to -0.775 | 0.0023 | 1.80 to 3.97 | < 0.0001 | 3.83 to 6.18 | < 0.0001 |
| *VCAM1* | 6.20±0.23 | 7.71±0.15 | 5.38±0.21 | -2.53 to -0.48 | 0.0041 | 0.0041 to 1.65 | 0.0513 | 1.44 to 3.22 | < 0.0001 |
| *MMP9* | 5.19±0.13 | 11.00±0.46 | 7.60±0.44 | -7.87 to -3.75 | < 0.0001 | -4.06 to -0.75 | 0.0045 | 1.61 to 5.20 | 0.0004 |
| *IDO1* | 4.00±0.07 | 8.77±0.19 | 6.43±0.19 | -5.65 to -3.90 | < 0.0001 | -3.14 to -1.73 | < 0.0001 | 1.58 to 3.10 | < 0.0001 |

S5-Table 2. The expression level of 6 hub genes for the different phases of UC-related carcinogenesis in GSE31106.

|  | Control | UC | UC-Cancer | Control vs UC | | Control vs Adenocarcinoma | | UC vs Adenocarcinoma | |
| --- | --- | --- | --- | --- | --- | --- | --- | --- | --- |
|  | (Mean±SE) | (Mean±SE) | (Mean±SE) | 95% CI | Adj. P | 95% CI | Adj. P | 95% CI | Adj. P |
| Ccr7 | 6.51±0.17 | 9.50±0.21 | 7.37±0.35 | -4.11 to -1.88 | 0.0004 | -1.98 to -0.25 | 0.012 | 1.01 to 3.24 | 0.0027 |
| Cxcl10 | 6.13±0.03 | 8.58±0.7 | 7.43±0.17 | -4.45 to -0.45 | 0.0221 | -3.30 to -0.70 | 0.020 | 0.85 to 3.15 | 0.026 |
| Cxcl9 | 6.40±0.34 | 8.17±0.27 | 5.67±0.09 | -2.91 to -0.64 | 0.0072 | 0.41 to 1.86 | 0.020 | 1.37 to 3.64 | 0.0012 |
| Vcam1 | 8.04±0.16 | 9.44±0.10 | 7.49±0.01 | -1.87 to -0.93 | 0.0002 | 0.076 to 1.02 | 0.028 | 1.478 to 2.4 | < 0.0001 |
| Mmp9 | 5.13±0.10 | 8.77±0.62 | 6.58±0.14 | -5.25 to -2.03 | 0.0011 | -3.06 to -0.16 | 0.007 | 0.58 to 3.80 | 0.014 |
| Ido1 | 5.05±0.46 | 8.38±0.26 | 6.73±0.17 | -4.73 to -1.93 | 0.0008 | -3.08 to -0.28 | 0.024 | 0.25 to 3.04 | 0.026 |
