# Supplementary material for: Analysis of Genes Involved in Ulcerative Colitis Activity and Tumorigenesis Through Systematic Mining of Gene Co-expression Networks
Source: Front Physiol. 2019 May 31;10:662. doi: 10.3389/fphys.2019.00662 (PMC6554330; doi:10.3389/fphys.2019.00662)
Supplement: Supplementary file 7 [file Image_1.pdf]

Figure S1. The supplement of the weighted correlation network construction.

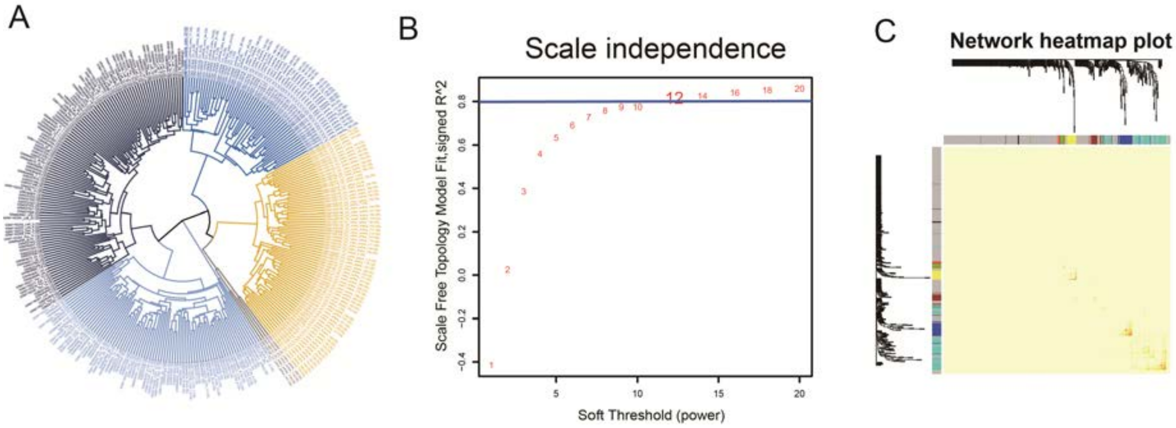

The construction of the weighted correlation network. A) cluster analysis of samples based on the flashClust method. B) The soft thresholding selection. C) Interactions analysis among gene co-expression modules. The brightness of the yellow color in the middle represents the relativity degree of different modules.
